# Effect of piezocision procedure in levelling and alignment stage of fixed orthodontic treatment: a randomized clinical trial

**DOI:** 10.1038/s41598-022-09851-0

**Published:** 2022-04-14

**Authors:** Sharmin Sultana, Norma Ab Rahman, Siti Lailatul Akmar Zainuddin, Basaruddin Ahmad

**Affiliations:** 1 Manipal University College Malaysia, Jalan Batu Hampar, Bukit Baru, 75150 Melaka, Malaysia; 2 Orthodontics Unit, School of Dental Sciences, Health Campus, Universiti Sains Malaysia, 16150 Kubang Kerian, Kelantan Malaysia; 3 Periodontics Unit, School of Dental Sciences, Health Campus, Universiti Sains Malaysia, 16150 Kubang Kerian, Kelantan Malaysia; 4 Dental Public Health (Biostatistics) Unit, School of Dental Sciences, Health Campus, Universiti Sains Malaysia, 16150 Kubang Kerian, Kelantan Malaysia

**Keywords:** Biotechnology, Health care, Medical research

## Abstract

This clinical trial compared the time to complete the levelling and alignment stage with flapless piezocision procedure in the treatment of severe maxillary malocclusion with premolar extraction cases. Two-arm parallel group randomized controlled trial was performed at the Orthodontics Unit of Universiti Sains Malaysia, Malaysia. Sixteen patients with severe anterior maxillary crowding (Little’s irregularity index: 7–9 mm) and required bilateral first premolars extraction was recruited. The participants were randomly assigned to a study group according to a simple randomization method using a sealed envelope mentioned about the group name. Both groups were treated with fixed orthodontic appliance using the 0.022-in. slot of McLaughlin Bennett Trevisi prescription brackets. The piezocision group received flapless piezocision corticotomy about 4–5 mm in length and 3 mm depth on the labial mucogingiva between the roots of six anterior teeth. The number of days since treatment started, Little’s irregularity index, gingival recession, pocket depth, pulp vitality, patient perception of the pain and satisfaction level were recorded before the treatment, at about 1 month and 2 months post-treatment, and at the completion of the levelling and alignment stage. The overall time to complete levelling and alignment stage was significantly shorter in the piezocision group than the control group (mean difference = 31.5 days, 95% CI 6.5, 56.5; p = 0.018). Greater reduction in Little’s irregularity index and faster alignment rate in the first 2 months  were found in the piezocision group compared to the control group (p < 0.05). No changes in the gingival recession, pocket depth, and pulp vitality in both groups were observed. Patients who received piezocision surgery experienced no or mild pain and were satisfied with the treatment. Flapless piezocision corticotomy is an effective adjunct that shortens treatment time during levelling and alignment stage without any adverse effects on the teeth and surrounding tissues. It is also painless, acceptable and satisfactory to the patients.

**Trial registration:** ACTRN12621001350819.

## Introduction

Dental crowding is one of the common malocclusion in the population and conventionally treated using fixed orthodontic appliances^1^. In severe and complex crowding cases where there is lack of space to achieve a good alignment, for instance, in those with Little’s irregularity index (LII) greater than 7 mm, bilateral extraction of the first premolars are indicated^1,2^. In such cases, more teeth are moved and thus, greater overall tooth movement and distance; hence, the time to complete the treatment is likely to be longer and involves multiple appointments.

Various non-surgical and surgical methods to accelerate orthodontic tooth movement (OTM) and shorten treatment time has been introduced following the discovery of the regional acceleratory phenomenon (RAP)^3^, a bone healing and remodelling response following an injury characterized by increased osteoclastic and osteoblastic activities^4^. Examples are the periodontally accelerated osteogenic orthodontics, corticotomies, osteotomies, corticision, piezocision, perforation, puncture and discision^5^. Earlier corticotomy methods that require a full-thickness mucoperiosteal flap are effective but they traumatise the supporting tissues. More recent flapless techniques are less traumatic, allow quicker soft tissue healing at the site of corticotomy and lower the risk of infection^6^. Moreover, patient satisfaction and acceptance about the piezicision procedure also higher even though they feel pain after the piezocision surgery^7^.

Piezocision is one of the flapless corticotomy methods. The results of earlier studies using the procedure on moderate crowding without premolar extraction cases^8–11^, for canine or en-masse retraction^12–15^ are mixed; seven trials showed a significant reduction in treatment time favouring the piezocision^1,8–10,12–14^, and two trials showed no advantage^11,15^. The study conducted by Uribe et al. did not show significant finding and the protocol was different compared to other studies. The authors had performed the corticotomy at 1mm depth and only in 3 sites of the mandible^11^; it has suggested that greater corticotomy depth increases the RAP effect thereby accelerate tooth movement^3,4^. A recent systematic review suggested that the procedure is effective for canine retraction based on low-quality evidence but the evidence is not clear for en-masse retraction^16^.

Malocclusion caused by moderate to severe crowding is more complex and often require bilateral premolar tooth extraction to manage the space^1^. Thus far, only one trial investigated the effect of the procedure in such cases and found that it shortens the overall treatment time to complete the levelling and alignment of lower anterior teeth by 59% compared to the traditional method^1^. Currently, no study had investigated the effect of piezocision on severe crowding case in the maxillary arch. Thus, the primary objective of this randomized clinical trial was to compare the time to complete levelling and alignment stage between the piezocision procedure and conventional orthodontic treatment in patients with severe crowding based on LII of anterior maxillary teeth who required bilateral first premolar extraction. The secondary objectives were to investigate the effect of piezocision on surrounding tooth structure and patients satisfaction with the procedure. This study hypothesized that flapless corticotomy using piezocision procedure could accelerate the tooth movement and reduce the treatment time of levelling and alignment stage than conventional orthodontic treatment. In addition, this approach is expected to result in minimal changes to the surrounding tooth structures, which are comparable to the conventional orthodontic method, with limited pain experience and optimum satisfaction in the piezocision group.

## Materials and methods

### Study design

This two-arm parallel-group randomized clinical trial was carried out at the Specialist Orthodontic Clinic, Universiti Sains Malaysia.

### Ethical consideration

Ethical approval has been obtained from the review board of the institution of Universiti Sains Malaysia (USM) No: (USM/JEPeM/17110591). All methods were performed in accordance with the relevant guidelines and regulations. Written informed consent was obtained before the patients were randomized into the treatment groups. The trial was registered with Australian New Zealand Clinical Trials Registry (ANZCTR) with the trial number of ACTRN12621001350819.

### Sample size calculation

Sample size calculation was performed using the online calculator^17^, assuming that a minimum of four (4) weeks (28 days) difference in treatment time between the two treatment arms is beneficial to the patients and clinicians. Using the SD = 14.18 days from a study to correct the levelling and alignment in severe maxillary incisor crowding^2^, power = 0.9 and significance level = 0.05, the calculation showed that six patients per arm were required in this study. Considering a drop-out rate of 30%, the study had recruited eight (8) patients per group (N = 16).

### Patients selection

Patients were selected among those who attended the Specialist Orthodontic Clinic at Hospital Universiti Sains Malaysia for assessment of orthodontic treatment. All routine orthodontic diagnostic records such as study models, orthopantomograms, lateral cephalograms, extraoral and intraoral photographs, gingival recession and pocket depth were taken for initial pre-treatment record, diagnostic purposes and treatment plan. Patients who met the inclusion and exclusion criteria were invited to participate in the study. The inclusion criteria were; (1) adults aged between 18 and 30 years, (2) clinically healthy without systemic disease or medical condition, (3) have LII of 7–9 mm in the anterior maxilla and require bilateral extraction of the first premolars, (4) feasible to bond the brackets and engage the initial archwire into the brackets of all the bonded maxillary teeth, and perform the piezocision procedure on the experimental group on same day, (5) vital teeth and periodontal tissues with probing depth < 3 mm across the entire dentition and less than 2 mm clinical attachment loss, and (6) no radiographic evidence of root resorption. The exclusion criteria were: (1) syndromic craniofacial deformities, (2) taking medication such as NSAID’s, bisphosphonates or corticosteroids medications throughout the study period, (3) had a history of previous orthodontic treatment, and (4) had missing or impacted permanent teeth except for the third molar.

From a total of 187 patients screened between April 2018 to December 2019, 156 did not meet the criteria and 15 had declined from participating. Sixteen patients who met the inclusion and exclusion criteria were recruited over the stated period and briefed about the purpose of study, conventional orthodontic treatment, piezocision procedure, and the risks and benefits of participation.

### Randomization and treatment allocation

Random allocation and allocation concealment was performed by preparing 16 sealed and unnumbered envelopes containing a piece of paper printed with the word ‘Piezocision’ or ‘Conventional’. Each consented patient was asked to select one envelope and opened it in front of the researcher (S.S.) for the record. This approach prevents selection bias in this unblinded trial and randomly assigns the patients to the treatment groups.

### Orthodontic procedures

Both groups were treated with conventional fixed orthodontic appliance following seven to ten days after bilateral first premolar extraction. Pre-adjusted edgewise McLaughlin Bennett Trevisi prescription metal brackets and buccal tube (Natural orthodontic product) of the 0.022-in. slot were bonded from the right first molar to the left first molar. In piezocision group bonding of brackets and activation of initial arch wire was performed on same surgery day. To initiate the levelling and alignment phase of both groups, a 0.012-in. nickel-titanium archwire (3M Unitek) was inserted. Following, the archwire sequences 0.016-in., 0.017 × 0.025-in., and 0.019 × 0.025-in. nickel-titanium were used for ideal alignment. Each number of archwires were replaced only when the possibility for full engagement into the brackets slot with a minimal amount of bending and without expressing excessive force on anterior tooth movement^2^. The patients were evaluated every 4–6 weeks^11^. The levelling and alignment stage was considered as complete when the LII of the anterior teeth is 0 mm and the final archwire 0.019 × 0.025 ss can be passively inserted into all brackets.

### Piezocision procedure

The piezocision procedure followed that described in Dibart et al.^3^, but the subsequent follow-up visits were modified to 4–6 weeks instead of every 2 weeks originally. Before the surgical procedure started, patients rinsed the mouth with 0.2% chlorhexidine for 1 min. Then, using the panoramic radiograph as a guide, the root proximity and long axis of the teeth were determined and seven corticotomy lines were marked on the mucogingiva using a marker pen. Each line started at 3 mm away from the interdental papilla to preserve the papillary gingival margin and alveolar crest and extended upwards to about 4–5 mm in length. Next, local anaesthetic was administered using 2% Lidocaine with 1:100,000 epinephrine. After anaesthesia was achieved, an incision was made using 15C surgical scalpel blade through the gingiva on each of the marked lines at 45°–60° to the long axis of the teeth. The gingiva was then slightly elevated laterally to check the alveolar cortical bone and root. Piezocision was not performed in cases where there was root proximity^3^.

**Figure 1:**
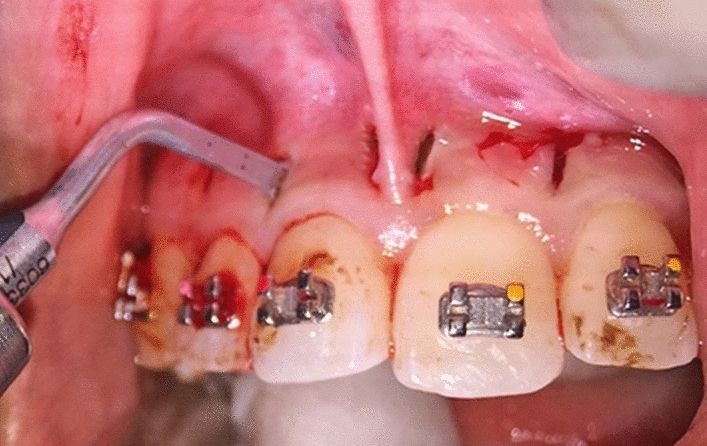
Piezocision flapless corticotomy procedure performed on maxillary anterior teeth. The surgical blade no 1 with millimeter marker showed 3 mm depth of corticotomy.

**Figure 2:**
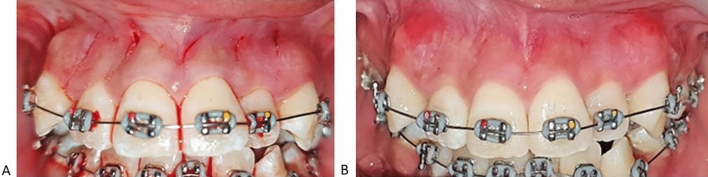
Piezocision flapless corticotomy procedure (**A**) Immediate after piezocision surgery, (**B**) Healing response after 1 week of surgery.

Cortical alveolar bone incisions were made using a piezosurgery blade (BS1 insert, Satelec Acteon Group) and it was positioned at the same angulation as the scalpel blade to make a 3 mm depth cut into the medullary bone. The depth of the corticotomy cut was determined using the marking on the BS1 blade and maintained throughout the corticotomy line. The piezotome device was set at low-frequency ultrasonic waves (28–36 kHz) for cutting cortical bone with a continuous 60% saline irrigation (Fig. 1). High-speed suction was used to remove excess fluid from the surgical area. Hemostasis was achieved using cotton gauze and thumb pressure. No sutures were placed after the procedure (Fig. 2). No bone grafting placement was performed. Post-operatively, the patients were advised to take analgesics (paracetamol) only when necessary, carefully brush the teeth and used 0.2% chlorhexidine mouthwash twice a day for one (1) week. All experimental subjects were advised to contact the Orthodontic Clinic of Hospital Universiti Sains Malaysia immediately if there was any complication after the surgery. All clinical procedure was carried out by one co-author (S.S) who is a qualified orthodontist.

### Primary and secondary outcome measures

Study outcomes were assessed at four time-points: before treatment (T0), 1-month post-treatment (T1), 2 months post-treatment (T2), and at the end of levelling and alignment stage (T3). Treatment time is defined as the number of days passed from T0. The primary outcome measure was the overall treatment time (T3-T0), defined as the total number of days to complete the alignment and levelling stage.

The secondary outcomes included the changes in LII (mm), alignment rate (mm/month), pocket depths, gingival recession, and pulp vitality, and patient’s perception of pain during the procedure and satisfaction with the piezocision procedure. LII assesses the severity of crowding and measured the total horizontal distance between two adjacent contact points of 6 anterior teeth^18^. The measurement was made using an electronic digital calliper to the nearest 0.01 mm on a dental cast taken at each time-point. The alignment rate at T1, T2 and T3 were the ratio of the difference in LII between two consecutive time points divided by the treatment time (per month) ($e.g. \; alignment \; rate \; at \; T2= \frac{{LII}_{T2}- {LII}_{T1}}{T2-T1}$). Gingival recession and pocket depth were assessed at T0 and T3 using a Williams periodontal probe (Hu Friedy, Chicago, III). Gingival recession was the distance from the cementoenamel junction to the free gingival margin at the labial site only. Pocket depth was recorded at three sites on the buccal side (mesiobuccal, buccal and distobuccal) to nearest 1 mm. The mean changes in gingival recession and pocket depth between T3 and T0 were calculated^19^.

### Assess of pulp vitality

Tooth vitality was recorded using an electric pulp tester (EPT) at T0 and T3. The teeth were isolated using cotton rolls, dried thoroughly, and applied with toothpaste before testing. The procedure was performed carefully to avoid contact between the tip of the tester and orthodontic wire, brackets, lips or tongue^20^. The gingival, periodontal and vitality status are also monitored at each visit.

### Patient’s perception of piezocision procedure

The experimental group completed a self-administered questionnaire that assessed the perception of pain and satisfaction with the procedure at the end of surgery and collected one (1) week later. The patients were asked whether they felt any pain after the surgery and to rate the response using a unidimensional measure that ranges from 0–10, which is then categorized as no pain (0), mild pain (1–3), moderate pain (4–6), severe pain (7–10). Satisfaction with the surgical procedure was assessed using the similar rating and the score was categorized as extremely unsatisfied (0), unsatisfied (1–2), neutral (3–5), satisfied (6–8), extremely satisfied (9–10)^21^.

### Statistical analysis

Descriptive analysis was carried out to summarize the sample and examine the data distribution. The t-tests and Chi-square test was used to compare the parameters between the control and experimental groups. Comparison between groups analyses followed the per-protocol principle. Comparison of the overall treatment time and changes in LII between the two groups were compared using independent t-test. The alignment rate was analysed using one-way repeated-measures analysis of variance. Significance level was set at 5%. Data were analyzed using SPSS version 26 (Chicago, USA).

### Reliability study

Reliability study was carried out by randomly selecting six dental casts and re-measure the LII one (1) month after the first assessment. The result showed strong intra-examiner reliability (intra-class coefficient, ICC = 0.99).

## Results

### Participant flow

**Figure 3:**
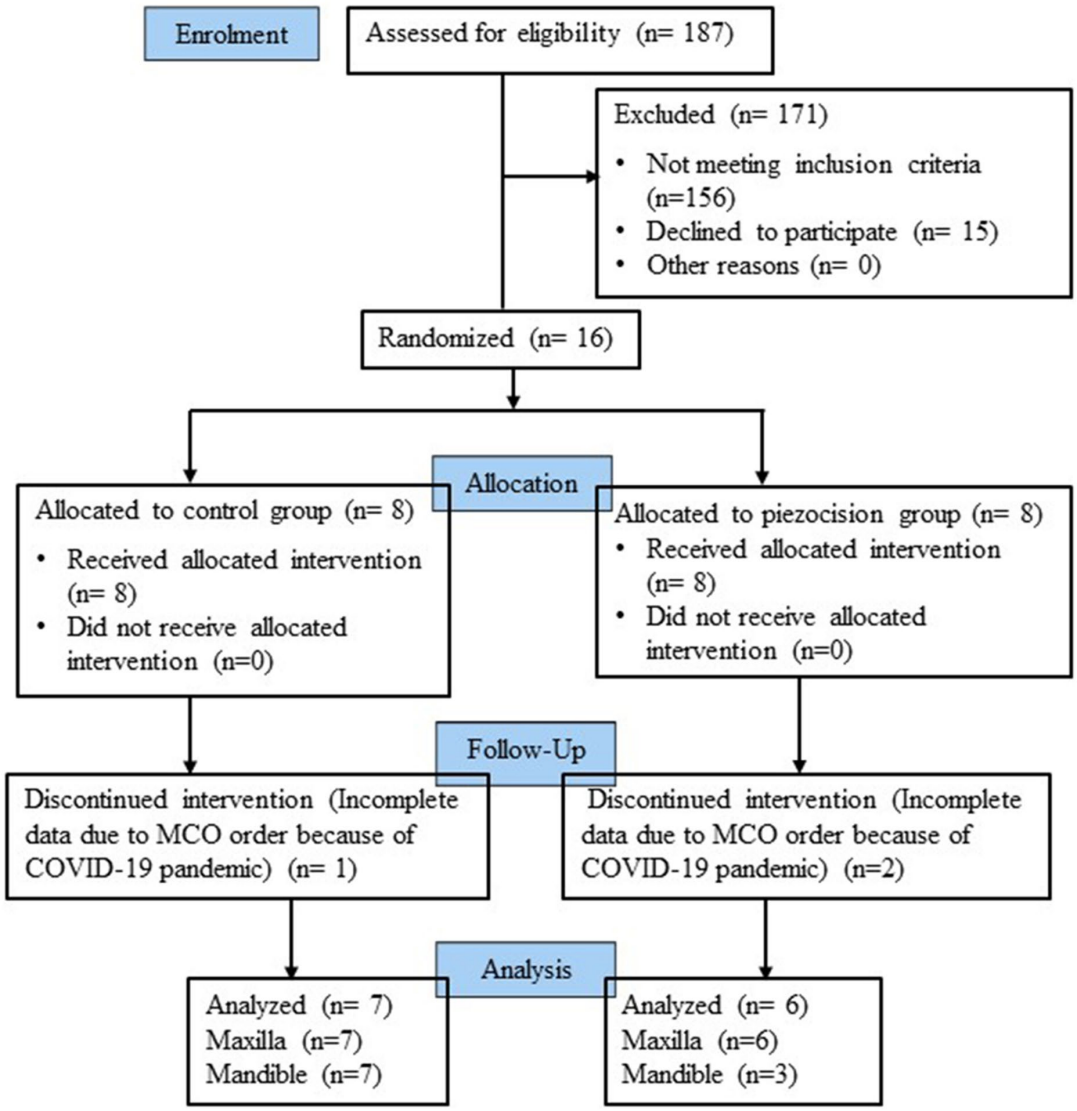
Consort diagram showing the overall patients flow throughout the clinical trial.

The patient flow through the trial using a CONSORT diagram is presented in Fig. 3. Sixteen patients were enrolled in the control and piezocision groups. All the patients received the allocated intervention. During the trial, the follow-up visit of one patient in the control, and two patients in the piezocision groups, whom all had received the allocated intervention, were halted because of movement control order by the Malaysian government due to coronavirus disease (COVID-19) pandemic. Consequently, continuous treatment (orthodontic appliance activation) and assessments that was planned at T1, T2 and T3 did not continue for these patients and their treatment resumed only after 5 to 6 months. Because the measurements no longer reflect the study protocol they were excluded from the analysis. The results of the analysis were from 7 and 6 patients in the control and experimental group, respectively.

### Patient characteristics

The characteristics of the study participants are presented in Table 1. The sample characteristics were similar in the two groups. The mean treatment time between T1-T0 and T2-T1 were similar in both group. However, treatment time between T3-T2 was significantly shorter in the piezocision group than the control group.

**Table 1:** Sample demographic and baseline.

Variables	Control group (n = 7)	Piezocision group (n = 6)	*p*
	(Mean ± SD)	(Mean ± SD)	
Age, years	21.14 ± 2.97	20.83 ± 2.32	0.8
**Gender, n (%)**			1.0
Female (n = 12)	6 (85.7%)	6 (100%)	
Male (n = 1)	1 (14.3%)	0 (0.0%)	
**T1-T0**			
Days	31.71 ± 5.77	32.33 ± 3.20	0.8
Month	1.04 ± 0.19	1.06 ± 0.11	0.8
**T2-T1**			
Days	28.57 ± 3.51	30.17 ± 4.07	0.4
Month	0.94 ± 0.12	0.99 ± 0.13	0.4
**T3-T2**			
Days	94.57 ± 23.80	60.83 ± 20.07	0.018*
Month	3.11 ± 0.78	2.00 ± 0.66	0.018*
**T3-T0**			
Days	154.86 ± 22.09	123.33 ± 18.23	0.018*
Month	5.09 ± 0.73	4.05 ± 0.60	0.018*

*Significant.

### Orthodontic outcomes

The analysis showed that the overall alignment time (T3-T0) in the piezocision group was shorter compared to the control group (mean difference = 31.5 days, p = 0.018) (Table 2) with 20.4% less number of days; the Hedges’ g effect size was 1.5^22^. The LII at T0 and T1 were comparable in both groups and the teeth were fully aligned (LII score = 0 mm) at T3 (end of levelling and alignment stage). The LII was significantly lower in the piezocision group at T2 (p < 0.01) (Table 2). Repeated measures ANOVA showed that the alignment rate was significantly higher at T1 and T2 compared to T3 (p < 0.01) (Appendix A), and in the piezocision than the control group (p < 0.009) (Table 2). The alignment rates were also significantly higher in the piezocision than the control at T1 and T2 (p < 0.05) (Table 2).

**Table 2:** Overall alignment time (in days), changes in Little’s Irregularity Index (LII) (mm), and changes in alignment rate (mm/month) between control and piezocision group.

Variables	Control group (n = 7)	Piezocision group (n = 6)	Mean difference (95%CI)	p-value
	(Mean ± SD)	(Mean ± SD)		
OAT (T3-T0)	154.86 ± 22.09	123.33 ± 18.23	31.52 (6.51 to 56.54)	0.018*
LII at T0	8.30 ± 0.68	8.31 ± 0.68	− 0.007 (− 0.84 to 0.82)	0.9
LII at T1	6.22 ± 0.73	5.54 ± 0.63	0.68 (− 0.16 to 1.52)	0.1
LII at T2	4.14 ± 0.76	2.89 ± 0.47	1.25 (0.46 to 2.04)	0.005*
LII at T3	0.00	0.00	–	–
AR at T1	− 2.09 ± 0.50	− 2.78 ± 0.60	0.57 (0.002 to 1.39)	0.049*
AR at T2	− 2.23 ± 0.35	− 2.70 ± 0.42	0.47 (0.009 to 0.939)	0.047*
AR at T3	− 1.37 ± 0.27	− 1.61 ± 0.66	0.24 (− 0.359 to 0.843)	0.3
OAR (T3-T0)	− 1.65 ± 0.15	− 2.09 ± 0.33	0.44 (0.136 to 0.743)	0.001^#^

OAT: Overall alignment time, LII: Littleˈs irregularity index, AR: Alignment rate, OAR: Overall alignment rate, *Alignment rate, ^#^ repeated measure ANOVA comparing the groups.

### Periodontal outcomes and tooth vitality test

No significant change in the pocket depth and clinical attachment was found between T3-T0 in both groups (p > 0.05) (Appendix B) and no teeth became non-vital during the study (Appendix C).

### Pain and satisfaction score in piezocision group

In the piezocision group, 6 (75%) had mild pain and 2 (25%) and no pain and, 5 (62.5%) were satisfied and 3 (37.5%) were extremely satisfied with the procedure (Appendix D).

## Discussion

This trial is the first to evaluate the time to complete levelling and alignment stage in patients with severe anterior maxillary crowding require bilateral extraction of the first premolar comparing the adjunct effect of flapless piezocision procedure to conventional treatment only. The results showed that patients who received the piezocision procedure completed the stage about a months earlier than the conventional method or about 20% less number of days. The difference in time to complete the stage in the present study was 59% less compared to other similar study conducted in severe crowding in the mandible^1^.

The Little’s irregularity index is observed to decrease gradually during the alignment phase; there was no difference between the groups at 1-month post-treatment but was significantly different at 2 months post-treatment. That observation somewhat concurs with the alignment rate, whereby it was higher in the piezocision group at the first two treatment periods (T0 to T1 and T1 to T2). The difference was, however, similar in the last period (T2 to T3). This pattern is similarly observed reported in the study on the mandible^1^.

These observations are consistent with the understanding of the RAP phenomenon. The injury from the piezocision corticotomy induces wound healing responses in the alveolar bone and periodontal tissues and increases the formative (fibroblasts, cementoblasts, and osteoblasts) and resorptive (osteoclasts) cells activity, which in turn enhances bone remodelling and accelerate OTM^23–26^. The effect of RAP is reported to lasts between 2 to 4 months after the induction and decrease gradually over time^24–26^. The magnitude of the RAP effect on tooth movement is highly dependent on the invasiveness of the surgical procedure^3,4^.

There are five main differences that possibly explain the slower time to complete the levelling and alignment stage in the present study compared to the study on the mandible. First, although both studies used the same depth of cut, the longer length of cut (5-8mm) in the study on mandible, compared to 4-5mm in the present study, triggers a greater (area of) RAP response. Second, tooth movement started earlier in mandible study^1^; it is activated 1 week before the surgery compared to the same day as piezocision surgery in the present study. Third, activation of tooth movement is more frequent in the mandible study^1^ than the current study (twice a month vs once a month); more frequent activation within the optimum active phase of RAP, is expected to yield greater movement rate. Fourth, tooth movement in the maxilla is relatively slower than the mandible because the maxillary teeth are generally larger in size. It is also influenced by the severity of crowding, bone thickness and patient's age. The use of LII, which measures crowding based on horizontal distance, does not reflect the changes at molecular level taking place during tooth movement. Fifth, the earlier study completed the levelling and alignment when the LII is less than 1 mm, which is likely to be earlier than the present study which defined the completion at 0 mm.

The overall treatment time in this study is comparable to another non-surgical method using the low-level laser therapy for the treatment of malocclusion cases with similar severity (mean difference = 28 days compared to control)^2^. Besides the difference in the instruments used to induce RAP, the laser study recalled the patients on days 3, 7, and 14 after the first laser irradiation and every 15 days from the second month until the levelling and the alignment stage is completed. The study, however, also considered the levelling of the maxillary anterior teeth is completed at LII less than 1 mm. The laser procedure coupled with a higher frequency of appliance activation does not show a higher rate of tooth movement than the present study.

The piezocision procedure does not cause any adverse effect on tooth vitality, periodontal tissues or other surrounding structures in this clinical trial, consistent with earlier corticotomy studies using similar or different methods^9,10,27^. The post-operative pain was mild and patients did not require analgesic. There was also high acceptance and satisfaction level among the patient who received piezocision.

## Limitations

Findings from this study should be interpreted with caution. Although the small sample size of this study is small compared to earlier reports and having lost three participants after treatment and intervention was carried out due to unfortunate and unavoidable circumstances, the number met the minimum requirement calculated at the beginning of the study and thus, maintained the study power. Despite the absence of any clinical adverse effects, there was no radiographic assessment to examine potential changes such as root resorption, bone density, healing of corticotomy. Randomised selection of cases cannot be done due to limited availability of sample that met the inclusion and exclusion criteria. Blinding of treatment allocation is not applicable in this study and has been adequately addressed using the sealed envelope randomization method.

## Conclusion

The modified piezocision procedure significantly reduces the time for levelling and alignment of severe crowding of the anterior maxillary teeth without any adverse effect. The high rate of tooth movement is noticeable in the first 2 months after inducing the RAP.

## Supplementary Information

Supplementary Information 1.

Supplementary Information 2.

Supplementary Information 3.

Supplementary Information 4.

## Data Availability

The data underlying this article will be shared upon reasonable request to the corresponding authors.

## Abbreviations

LII: Little’s irregularity index
OTM: Orthodontic tooth movement
RAP: Regional acceleratory phenomenon
EPT: Electric pulp tester
