# Supplementary material for: Effect of piezocision procedure in levelling and alignment stage of fixed orthodontic treatment: a randomized clinical trial
Source: Sci Rep. 2022 Apr 14;12:6230. doi: 10.1038/s41598-022-09851-0 (PMC9008391; doi:10.1038/s41598-022-09851-0)
Supplement: Supplementary file 1 — Supplementary Information 1. [file 41598_2022_9851_MOESM1_ESM.docx]

**Appendix A**

**1. One way repeated measure ANOVA**

| Alignment rate | Total sample | *P* value (rate) | *P* value  (group) |
| --- | --- | --- | --- |
|  | (Mean ±SD) |  |  |
| T1-T0 | -2.30± 0.53 | <0.001* | 0.001* |
| T2-T1 | -2.45± 0.44 |  |  |
| T3-T2 | -1.45± 0.49 |  |  |

**2. Post hoc comparison for time and group**

| Alignment rate and group | Mean difference (95% CI) | Std. error | *P* value |
| --- | --- | --- | --- |
| T1-T0 - T2-T1 | 0.15 (- 0.26 to 0.56) | 0.19 | 0.4 |
| T1-T0 - T3-T2 | - 0.83 (- 1.31 to - 0.35) | 0.22 | 0.003* |
| T2-T1- T3-T2 | - 0.98 (- 1.38 to - 0.57) | 0.18 | <0.001* |
| Piezocision - Control | - 0.43 (- 0.65 to - 0.21) | 0.10 | 0.001* |
