# Supplementary material for: Effect of piezocision procedure in levelling and alignment stage of fixed orthodontic treatment: a randomized clinical trial
Source: Sci Rep. 2022 Apr 14;12:6230. doi: 10.1038/s41598-022-09851-0 (PMC9008391; doi:10.1038/s41598-022-09851-0)
Supplement: Supplementary file 2 — Supplementary Information 2. [file 41598_2022_9851_MOESM2_ESM.docx]

**Appendix B**

**Changes in pocket depths and gingival recession (mm) between control and piezocision group.**

| Variables | | Control group (n=7) | | | | Piezocision group (n=6) | | | | *P* value |
| --- | --- | --- | --- | --- | --- | --- | --- | --- | --- | --- |
|  |  |  | (Mean±SD) | Median | IQR |  | (Mean±SD) | Median | IQR |  |
| Pocket depths | Baseline T0 |  | 1.55±  0.24 | 1.43 | 0.30 |  | 1.60 ± 0.28 | 1.48 | 0.57 | 0.9 |
|  | After alignment T3 |  | 1.62 ± 0.28 | 1.50 | 0.57 |  | 1.67 ± 0.27 | 1.62 | 0.54 | 0.8 |
|  | Changes  (T3-T0) |  | 0.08 ± 0.12 | 0.07 | 0.11 |  | 0.07 ± 0.06 | 0.06 | 0.12 | 0.9 |
| Gingival recession | Baseline T0 |  | 0.07±  0.09 | 0.06 | 0.13 |  | 0.13± 0.16 | 0.07 | 0.28 | 0.7 |
|  | After alignment T3 |  | 0.08 ± 0.28 | 0.06 | 0.13 |  | 0.15 ± 0.15 | 0.13 | 0.28 | 0.4 |
|  | Changes  (T3-T0) |  | 0.01 ± 0.03 | 0.00 | 0.00 |  | 0.02 ± 0.05 | 0.00 | 0.03 | 0.8 |

*Non-parametric tests (Mann–Whitney U-tests) were conducted to evaluate the changes of pocket depths in each studied time point between the two groups.
