# Supplementary material for: Effect of piezocision procedure in levelling and alignment stage of fixed orthodontic treatment: a randomized clinical trial
Source: Sci Rep. 2022 Apr 14;12:6230. doi: 10.1038/s41598-022-09851-0 (PMC9008391; doi:10.1038/s41598-022-09851-0)
Supplement: Supplementary file 3 — Supplementary Information 3. [file 41598_2022_9851_MOESM3_ESM.docx]

**Appendix C**

**EPT responses of maxillary teeth between control and piezocision group.**

| Variables | Control group (n=7) | | | | Piezocision group (n=6) | | | *P* value |
| --- | --- | --- | --- | --- | --- | --- | --- | --- |
|  | Teeth number | (Mean±SD) | Median | IQR | (Mean±SD) | Median | IQR |  |
| EPT at T0 | 11 | 8.86 ± 1.77 | 10 | 3.00 | 9.33± 1.75 | 9.50 | 2.75 | 0.6 |
|  | 12 | 9.14±1.07 | 9.00 | 1.00 | 8.17±1.60 | 8.00 | 3.25 | 0.2 |
|  | 13 | 11.29±1.98 | 11.00 | 3.00 | 11.33±1.51 | 11.00 | 2.50 | 0.9 |
|  | 15 | 11.00±2.31 | 11.00 | 3.00 | 9.83±2.99 | 10.00 | 5.75 | 0.4 |
|  | 16 | 10.57±1.81 | 10.00 | 1.00 | 8.83±1.83 | 9.00 | 2.75 | 0.1 |
|  | 21 | 7.71±2.21 | 7.00 | 4.00 | 8.67±1.51 | 8.00 | 2.50 | 0.3 |
|  | 22 | 8.43±1.51 | 8.00 | 1.00 | 7.50±1.38 | 7.00 | 1.75 | 0.2 |
|  | 23 | 11.14±2.19 | 12.00 | 2.00 | 11.33±1.51 | 12.00 | 2.50 | 0.8 |
|  | 25 | 10.00±2.89 | 11.00 | 6.00 | 9.17±2.23 | 9.00 | 3.75 | 0.5 |
|  | 26 | 10.14±2.67 | 10.00 | 3.00 | 9.50±1.76 | 9.00 | 2.75 | 0.6 |
| EPT at T3 | 11 | 8.14 ± 2.12 | 8.00 | 5.00 | 7.67 ± 1.03 | 8.00 | 1.50 | 0.6 |
|  | 12 | 7.57±1.90 | 8.00 | 2.00 | 8.00±0.89 | 8.00 | 2.00 | 0.6 |
|  | 13 | 10.86±1.86 | 12.00 | 2.00 | 10.33±1.03 | 10.00 | 1.50 | 0.5 |
|  | 15 | 8.86±2.85 | 8.00 | 4.00 | 9.83±1.47 | 9.50 | 2.50 | 0.4 |
|  | 16 | 9.57±1.62 | 9.00 | 2.00 | 10.33±2.07 | 11.00 | 3.50 | 0.4 |
|  | 21 | 7.43±2.44 | 8.00 | 5.00 | 8.83±1.72 | 8.50 | 2.00 | 0.2 |
|  | 22 | 7.86±1.77 | 8.00 | 3.00 | 8.83±1.83 | 9.00 | 2.75 | 0.3 |
|  | 23 | 10.00±2.45 | 10.00 | 4.00 | 9.67±1.03 | 10.00 | 1.50 | 0.7 |
|  | 25 | 9.86±3.02 | 12.00 | 5.00 | 9.83±1.33 | 10.00 | 1.75 | 0.9 |
|  | 26 | 9.86±1.21 | 10.00 | 1.00 | 10.50±2.17 | 10.00 | 3.75 | 0.5 |
