# Supplementary material for: Effect of piezocision procedure in levelling and alignment stage of fixed orthodontic treatment: a randomized clinical trial
Source: Sci Rep. 2022 Apr 14;12:6230. doi: 10.1038/s41598-022-09851-0 (PMC9008391; doi:10.1038/s41598-022-09851-0)
Supplement: Supplementary file 4 — Supplementary Information 4. [file 41598_2022_9851_MOESM4_ESM.docx]

**Appendix D**

**Frequency distribution of pain and satisfaction score in the piezocision group.**

| Variables Pain score | | Frequency Percentage | |
| --- | --- | --- | --- |
|  |  |  |  |
| Pain score | No pain (0) | 2 | 25 |
|  | Mild pain (1-3) | 6 | 75 |
|  | Moderate pain (4-6) | 0 | 0 |
|  | Severe pain (7-10) | 0 | 0 |
|  | Total | 8 | 100 |
| Satisfaction score | Extremely unsatisfied (0) | 0 | 0 |
|  | Unsatisfied (1-2) | 0 | 0 |
|  | Neutral (3-5) | 0 | 0 |
|  | Satisfied (6-8) | 5 | 62.5 |
|  | Extremely satisfied (9-10) | 3 | 37.5 |
|  | Total | 8 | 100 |
